# Development and validation of prediction models for risk of adverse outcomes in women with early-onset pre-eclampsia: protocol of the prospective cohort PREP study

**DOI:** 10.1186/s41512-016-0004-8

**Published:** 2017-02-20

**Authors:** John Allotey, Nadine Marlin, Ben W. Mol, Peter Von Dadelszen, Wessel Ganzevoort, Joost Akkermans, Asif Ahmed, Jane Daniels, Jon Deeks, Khaled Ismail, Ann Marie Barnard, Julie Dodds, Sally Kerry, Carl Moons, Khalid S. Khan, Richard D. Riley, Shakila Thangaratinam

**Affiliations:** 1 Women’s Health Research Unit, Barts and the London School of Medicine and Dentistry, Queen Mary University of London, London, UK; 2 Pragmatic Clinical Trials Unit, Barts and the London School of Medicine and Dentistry, Queen Mary University of London, London, UK; 3 Multidisciplinary Evidence Synthesis Hub (MESH), Queen Mary University of London, London, UK; 4 The Robinson Research Institute, School of Paediatrics and Reproductive Health, University of Adelaide, Adelaide, Australia; 5 Institute of Cardiovascular and Cell Sciences, St George’s, University of London, London, UK; 6 Department of Obstetrics and Gynaecology, Academic Medical Centre, Amsterdam, The Netherlands; 7 Department of Obstetrics, Leiden University Medical Center, Leiden, The Netherlands; 8 Aston Medical School, Aston University, Birmingham, UK; 9 University of Birmingham Clinical Trials Unit, Edgbaston, Birmingham, UK; 10 School of Health and Population Sciences, University of Birmingham, Birmingham, UK; 11 Birmingham Centre for Women’s and Children’s Health, University of Birmingham, Birmingham, UK; 12 Action on Pre-eclampsia (APEC) Charity, Evesham, Worcestershire UK; 13 Julius Centre for Health Sciences and Primary Care, University Medical Centre Utrecht, Utrecht, The Netherlands; 14 Research Institute of Primary Care and Health Sciences, Keele University, Keele, UK

**Keywords:** Early-onset pre-eclampsia, Prognosis, Prediction model, Complication risk, Maternal, Fetal

## Abstract

**Background:**

Early-onset pre-eclampsia with raised blood pressure and protein in the urine before 34 weeks’ gestation is one of the leading causes of maternal deaths in the UK. The benefits to the child from prolonging the pregnancy need to be balanced against the risk of maternal deterioration. Accurate prediction models of risks are needed to plan management.

**Methods:**

We aim to undertake a multicentre prospective cohort study (Prediction of Risks in Early onset Pre-eclampsia (PREP)) to develop clinical prediction models in women with early-onset pre-eclampsia, for risk of adverse maternal outcomes by 48 h and by discharge. We will externally validate the models in two independent cohorts with 634 and 216 women. In the secondary analyses, we will assess risk of adverse fetal and neonatal outcomes at birth and by discharge.

**Discussion:**

The PREP study will quantify the risk of maternal complications at various time points and provide individualised estimates of overall risk in women with early-onset pre-eclampsia to plan the management.

**Trial registration:**

ISRCTN registry, ISRCTN40384046

## Background

Pre-eclampsia is a multisystem disorder in pregnancy associated with hypertension and proteinuria [1–3] and occurs before 34 weeks in 1% of women, so called early-onset pre-eclampsia [4, 5]. In the UK, hypertensive disease in pregnancy remains a leading cause of direct maternal deaths and contributes to about 20% of all stillbirths [6].

With an increased risk of maternal complications and 20-fold higher maternal mortality, early-onset pre-eclampsia is considered to be pathophysiologically different from the late-onset disease [7–9]. The only known cure is delivery of the baby and placenta. However, fetal and neonatal benefits from delaying the pregnancy need to be balanced against the risk of multisystem dysfunction in the mother.

Although the proportion of women with early-onset pre-eclampsia is only 1% of all pregnancies, the complexity of the treatment gives rise to large health care costs [4, 5]. Women are often admitted to tertiary care, and with a third experiencing complications, a stay in an intensive care facility may become necessary [10]. Infants also have longer stays in intensive care facilities for management of complications such as lifelong handicaps arising as a result of prematurity.

One of the key recommendations in the last CEMACH (Confidential Enquiries into Maternal and Child Health) (now known as Centre for Maternal and Child Enquiries (CMACE)) report is the need to adopt an early warning system to help in the timely recognition, referral and treatment of women who have or are developing critical conditions [6]. Early identification of mothers with early-onset pre-eclampsia at risk of complications, and their risks for these at various time points after diagnosis, will allow clinicians to make decisions on commencing interventions such as administration of steroids and, if needed, in utero transfer to a tertiary unit. In mothers considered to be of low risk, they can be monitored as an outpatient, and delivery may be delayed to lower the risk of perinatal complications from prematurity.

We aim to develop prediction models to quantify the overall risk of adverse maternal outcomes in women with early-onset pre-eclampsia and at various time points after diagnosis.

## Methods/design

The Prediction of Risks in Early onset Pre-eclampsia (PREP) study will be developed using existing recommendations on prognostic research model development and validation [11–13] and reported in line with the Transparent Reporting of a multivariable prediction model for Individual Prognosis Or Diagnosis (TRIPOD) statement [14]. Ethical approval for the study was received from the NRES Committee West Midlands (approval number 11/WM/0248), and the study was registered on ISRCTN registry as per the requirements set out by the World Health Organization (WHO) International Clinical Trials Registry Platform (ICTRP) and the International Committee of Medical Journal Editors (ICMJE) [15] guidelines with registration number ISRCTN40384046 [16].

### Objectives

The primary objectives are to develop and internally validate a prediction model in women admitted with early-onset pre-eclampsia from 20 weeks and 0 day to 33 weeks and 6 days of gestation, for assessment of the risk of adverse maternal outcomes at 48 h and by discharge. The 48 h of time interval was chosen to reflect the time recommended for delivery after administration of steroids to lower risks of respiratory distress in newborns. This time period is also considered to be the optimal duration to make decisions on timing and place of delivery and consider in utero transfer to a tertiary care unit if needed. The model will be externally validated in two independent datasets of patients with a diagnosis of early-onset pre-eclampsia. Our secondary objective is to assess the risk of adverse fetal and neonatal outcomes at birth and at any time until discharge.

### Study design and conduct

PREP is a prospective, multicentre, observational cohort study involving secondary and tertiary care obstetric units in England and Wales. All consecutive women with a suspected or confirmed diagnosis of pre-eclampsia before 34 weeks of gestation will be approached to take part in the study by research midwives and clinicians in antenatal clinics, wards, day assessment units and delivery suites. Clinicians managing the women will be requested to complete the Clinicians Management Plan at baseline to assess the reasons for planned management. The predictors evaluated in the PREP prediction model are routinely performed as part of standard clinical practice in women admitted with early-onset pre-eclampsia. The PREP study does not influence the management of these patients.

### Inclusion criteria

Women aged 16 years or over and a diagnosis of new-onset or superimposed pre-eclampsia at gestational age between 20 + 0 and 33 + 6 weeks will be considered for inclusion in the study. Women with a diagnosis of HELLP (haemolysis, elevated liver enzymes, low platelets) syndrome and those with one episode of eclamptic seizures without hypertension or proteinuria will be considered for inclusion. All women will be required to provide written informed consent and must be capable of understanding the information provided. The definitions for the diagnosis of pre-eclampsia are provided in Table 1.

**Table 1:** Definition of the inclusion criteria for recruitment into the PREP study

Condition	Definition
New-onset pre-eclampsia	New-onset hypertension (systolic BP ≥140 mmHg or diastolic BP ≥90 mmHg on 2 occasions 4–6 h apart in women) after 20 weeks of pregnancy and new-onset proteinuria (≥2+ in urine dipstick or PCR of greater than 30 mg/mmol or 300 mg of protein excretion in 24 h) [37]
Suspected pre-eclampsia	New-onset hypertension (systolic BP ≥140 mmHg or diastolic BP ≥90 mmHg on 2 occasions 4–6 h apart in women) after 20 weeks of pregnancy and 1+ proteinuria on urine dipstick
Superimposed pre-eclampsia	
- In women with chronic hypertension and no proteinuria before 20 weeks’ gestation	New-onset proteinuria (as defined previously)
- In women with significant proteinuria before 20 weeks’ gestation	Elevated serum alanine aminotransferase concentration (>70 U/L) or worsening hypertension (either 2 diastolic BP of at least 110 mmHg 4 h apart or 1 diastolic measurement of at least 110 mmHg if the woman had been treated with an anti-hypertensive drug), plus one of the following: increasing proteinuria, persistent severe headaches or epigastric pain
HELLP syndrome	Haemolysis, elevated liver enzymes, and low platelets syndrome: presence of haemolysis based on examination of the peripheral smear, elevated indirect bilirubin levels or low serum haptoglobin levels in association with significant elevation in liver enzymes and a platelet count below 100,000/mm^3^ after ruling out other causes of haemolysis and thrombocytopenia
1 episode of eclamptic seizures without hypertension or proteinuria	Other neurological conditions of seizures have been excluded

### Exclusion criteria

Women will be excluded if any of the outcomes (including recurrent eclamptic seizures) occurs prior to testing or if informed consent could not be obtained on time or if the mother does not have a good understanding of spoken and written English and a translator is unavailable.

### Candidate variables for inclusion in the model

Candidate predictor variables will be obtained from patient demographic characteristics and from clinical assessment including clinical history, examination and investigations. The predictors will be clearly defined and standardised to ensure reproducibility and enhance generalisability and application of study results to practice. Data will also be collected on any interventions applied between recruitment and outcome onset that may modify the outcome.

We will choose the candidate predictors a priori to be considered in the prediction model, based on the most promising predictor variables as identified with our previous systematic review [17–22]. The full list of variables to be evaluated for prediction of maternal and fetal outcomes is provided in Table 2.

**Table 2:** List of candidate predictor variables to be evaluated in the PREP study

Maternal characteristics Maternal age at diagnosis (years) Gestational age at diagnosis (weeks) Number of fetuses in pregnancy at time of consent (1, 2, 3)
History Summary score for medical history—1 point for each of the following: pre-existing hypertension, renal disease, diabetes mellitus, autoimmune disease, previous history of pre-eclampsia (0, 1, 2 or more)
Symptoms Headache and/or visual disturbance (yes/no) Epigastric pain, nausea and/or vomiting (yes/no) Chest pain and dyspnoea (yes/no)
Bedside examination and tests Systolic blood pressure (mmHg, highest measurement over 6 h) Diastolic blood pressure (mmHg, highest measurement over 6 h) Clonus (yes/no) Exaggerated tendon reflexes (yes/no) Abnormal oxygen saturation (<95% on air) (yes/no) Urine dipstick (0, 1+, 2+, 3+, 4+ or more)
Laboratory tests Haemoglobin (g/L) Platelet count (× 10^9^/L) ALT (IU/L) Serum uric acid (μmol/L) Serum urea (mmol/L) Serum creatinine (μmol/L) Urine protein-creatinine ratio (PCR) (mg/mmol)
Management at baseline Administration of oral and/or parenteral anti-hypertensives (ongoing or commenced within 1 day of diagnosis) (yes/no) Administration of magnesium sulphate (commenced before or within 1 day of diagnosis) (yes/no)
Additional factors for fetal model only Uterine artery Doppler (normal/abnormal) Liquor volume (normal/abnormal) CTG findings (normal/abnormal) Estimated fetal weight <10th centile (yes/no)

### Outcome measures

The primary outcome is a composite adverse maternal outcome including maternal neurological, haematological, cardiorespiratory, hepatic and renal complications and death. We prioritised the outcomes for clinical importance using a Delphi survey of researchers and clinicians. Prior to the analysis, after discussion with the independent Study Steering (SSC) and Data Monitoring (DMC) Committees, we included delivery before 34 weeks as a component of maternal composite outcome to minimise bias due to treatment paradox [23]. The secondary outcome is a composite adverse fetal outcome (Table 3).

**Table 3:** Components of the maternal and fetal composite outcomes

Outcome	Definition
Maternal outcomes	
Mortality	Maternal death at any time in pregnancy after delivery until discharge
Hepatic dysfunction	INR >1.2 indicative of DIC in the absence of treatment with warfarin. (DIC is defined as having both abnormal bleeding and consumptive coagulopathy (i.e. low platelets, abnormal peripheral blood film, or one or more of the following: increased INR, increased PTT, low fibrinogen, of increased fibrin degradation products that are outside normal non-pregnancy ranges).)
Hepatic haematoma or rupture	Blood collection under the hepatic capsule as confirmed by ultrasound or laparotomy
Glasgow coma score <13	From the GCS scoring system [39]
Stroke	Acute neurological event with deficits lasting longer than 48 h
Cortical blindness	Loss of visual acuity in the presence of intact papillary response to light
RIND	Cerebral ischaemia lasting longer than 24 h but less than 48 h revealed through clinical examination
Retinal detachment	Separation of the inner layers of the retina from the underlying RPE (choroid) and is diagnosed by ophthalmological examination
Acute renal insufficiency	For women with an underlying history of renal disease: defined as creatinine >200 μM; for patients with no underlying renal disease: defined as creatinine >150 μM
Dialysis	Including haemodialysis and peritoneal dialysis
Transfusion of blood products	Includes transfusion of any units of blood products: FFP, platelets, RBCs, cryo or whole blood
Positive ionotropic support	The use of vasopressors to maintain a systolic blood pressure >90 mmHg or mean arterial pressure >70 mmHg
Myocardial ischaemia/infarction	ECG changes (ST segment elevation or depression) without enzyme changes and/or any one of the following: (1) development of new pathologic Q waves on serial ECGs. The patient may or may not remember previous symptoms. Biochemical markers of myocardial necrosis may have normalised, depending on the length of time that has passed since the infarct developed. (2) Pathological findings of an acute, healed or healing MI. (3) Typical rise and gradual fall (troponin) or more rapid rise and fall (CK-MB) of biochemical markers of myocardial necrosis with at least one of the following: (a) ischaemic symptoms; (b) development of pathologic Q waves on the ECG; (c) ECG changes indicative of ischaemia (ST segment elevation or depression) or (d) coronary artery intervention (e.g. coronary angioplasty)
Require >50% oxygen for greater than 1 h	Oxygen given at greater than 50% concentration based on local criteria for longer than 1 h
Intubation other than for caesarean section	Intubation maybe by ventilation, electrical impedance tomography or continuous positive airway pressure
Pulmonary oedema	Clinical diagnosis with x-ray confirmation or requirement of diuretic treatment and SaO_2_ <94%
Postpartum haemorrhage	>1 L of blood loss after delivery
Early preterm delivery	Delivery at gestational age of less than 34 weeks
Fetal outcomes	
Perinatal or infant mortality	Death of a fetus or neonate. Infant mortality is the death of a child less than 1 year of age
Bronchopulmonary dysplasia	Oxygen requirement at 36 weeks’ corrected gestation unrelated to an acute respiratory episode
Necrotising enterocolitis including only Bell’s stage 2 or 3	Evidence of pneumotosis intestinalis on abdominal x-ray and/or surgical intervention
Grade III/IV intraventricular haemorrhage	Bleeding into the brain’s ventricular system, where the ventricles are enlarged by the accumulated blood or bleeding extends into the brain tissue around the ventricles
Cystic periventricular leukomalacia	Softening and necrosis in the hemispheric white matter in newborns that may result from impaired perfusion at the interface between the ventriculopetal and ventriculofugal arteries
Stage 3–5 retinopathy of prematurity	Abnormal blood vessel development in the retina of the eye, where blood vessel growth is severely abnormal, where there is a partially or totally detached retina
Hypoxic ischaemic encephalopathy	Apgar score ≤5 at 10 min and/or pH 7.00 in the first 60 min of life and/or base deficit ≥−16 in the first 60 min associated with an abnormal consciousness level (lethargy, stupor or coma) and seizures and/or poor/weak suck and/or hypotonia and/or abnormal reflexes

Adapted from the PIERS study

*INR* international normalised ratio, *DIC* disseminated intravascular coagulation, *PTT* partial thromboplastin time, *GCS* Glasgow Coma Scale, *RIND* reversible ischaemic neurologic deficit, *RPE* retinal pigment epithelium, *FFP* fresh frozen plasma, *RBCs* red blood cells, *cryo* cryoprecipitate, *ECG* electrocardiography

### Sample size considerations

From our systematic reviews [17], 20% of women with early-onset pre-eclampsia are expected to have adverse maternal outcomes at any time before discharge. Rules of thumb for fitting multivariate models suggest about 10 events for every variable are required to reduce issues with overfitting, and we will work within this constraint [24–26]. We initially planned to examine 10 predictor variables and hence planned to recruit 500 women. However, the event rate was lower than predicted, so we revised our plan to recruit sufficient women to obtain 100 events. Subsequent to the inclusion of delivery before 34 weeks as a component of the composite outcome, we revised our plan to include 22 candidate predictor variables, which was approved by the independent study Committees.

### Statistical analysis

We will develop two prediction models: a survival model for adverse maternal outcomes at various time points from diagnosis up to 34 weeks of gestation, including by 48 h after admission, and a logistic model for adverse maternal outcomes by discharge. These will both be developed using a general methodological and statistical framework as outlined below.

#### Model development, apparent performance assessment and internal validation

The predictors of adverse maternal outcomes will be identified to develop and externally validate a simple, interpretable and clinically applicable prediction model with face validity. We will use a transparent process that implements appropriate statistical methods and adheres to current methodological recommendations.

A backwards selection procedure will be used to decide which of the candidate predictor variables should be included in the final prediction model (with *p* < 0.15 taken conservatively to warrant inclusion). Continuous variables will be kept as continuous in the model (rather than dichotomising), to avoid a loss of power [27, 28]. Non-linear trends will also be considered using the multivariable fractional polynomial procedure [29, 30], which uses multivariable models to eliminate weaker predictors and identify transformations of continuous predictors that best predict outcome. Large amounts of missing variable data are not expected, but some will inevitably occur, with not all patients providing all variables of interest. Multiple imputation will be used to impute, with 5 imputations, under a missing at random assumption, missing values so as to avoid excluding patients from the analysis [27].

For survival analysis, failure event is defined as an adverse outcome or delivery occurring before 34 weeks of gestation. We will use a flexible parametric survival model via the Royston-Parmar approach [31–33], with the cumulative baseline hazard scale modelled using restricted cubic splines. The number of knots will be chosen by visual inspection. For the binary adverse maternal outcome by discharge, a logistic regression-modelling framework will be undertaken with the logit probability of an adverse outcome being the response variable.

We will assess the apparent performance of the fitted models for discrimination using the *C*-statistic (Harrell’s *C*-statistic from the survival model and AUC from the logistic model), with 95% confidence interval, averaging across the same imputed datasets that were used to generate the model. A *C*-statistic close to 1 indicates excellent discrimination and 0.5 indicates no discrimination beyond chance. The calibration performance of the model (fit of observed to expected risk across all individuals) will be assessed by checking whether the calibration slope is 1. As the model will be developed in the same data, perfect agreement is expected on average across the imputed datasets.

Non-parametric bootstrapping will be used to internally validate and examine the potential for overfitting of our developed models, by repeating the variable selection procedure in 100 bootstrap datasets from each of the 5 multiple imputation datasets, providing a total of 500 datasets. This leads to a new final model being produced in each of the bootstrap samples. We will average the difference in the performance of the models to obtain a single estimate of optimism for the *C*-statistic and the calibration slope. The optimism-adjusted performance statistics will be obtained by subtracting the above estimate of optimism from the original apparent performance statistics. The optimism-adjusted calibration slope will be taken as the uniform shrinkage, and the final models will be corrected by this shrinkage factor.

#### External validation

We will externally validate (as far as possible) the models in patients admitted with a diagnosis of early-onset pre-eclampsia in two prospective datasets: The Pre-eclampsia Eclampsia TRial Amsterdam (PETRA), Netherlands, and Pre-eclampsia Integrated Estimate of RiSk for mothers (PIERS), Canada. We will compare the predicted number of events from our model with the observed events in the external datasets to assess calibration for the logistic model and compare the predicted with the observed survival function for the survival model (as described above). We will also calculate the *C*-statistic curve to assess discriminatory ability.

The models we develop (which use data from women diagnosed with pre-eclampsia) will also be tested in women defined with suspected pre-eclampsia (urine dipstick 1+ on admission but normal 24-h proteinuria, <300 mg/24 h and normal PCR <30 mg/mmol). Such women will have been identified from our recruitment process, and by checking the calibration and discrimination of our models for such patients, we can examine the potential generalisability to a broader set of women.

## Discussion

Pre-eclampsia and its complications are still considered to be one of the leading causes of maternal and fetal morbidity and mortality; the condition utilises a large proportion of NHS resources. Accurate prediction and early management can significantly improve these outcomes. However, clinicians and policymakers currently do not have the evidence on which they can base their recommendations.

Timely prediction of complications in women with early-onset pre-eclampsia involves the use of a combination of maternal characteristics, symptoms, physical signs and investigations [21]. These ‘tests’ are to some extent haphazardly performed in all units, due to the absence of a structured approach. The most important determinant of perinatal outcome is gestational age, with more than half the chance of intact fetal survival when this is more than 27 weeks and the birth weight is more than 600 g [34].

A recent Cochrane review [10] has shown that expectant management reduced risk of fetal and neonatal complications without increase in maternal complications. Clinicians are reluctant to promote expectant management due to uncertainties around the scale of maternal risk. One of the main reasons for this lack of confidence in applying risk scores in practice is the absence of sufficient evidence to show the reproducibility and transportability of the model in a different population [11]. A good performing prediction model is one that is accurate, validated in populations and datasets external to those used to develop the model, widely applicable in practice, and acceptable to patients and ultimately improves clinical outcomes by helping clinicians and patients make more informed decisions. With very small numbers of cases and events per variable, early-onset pre-eclampsia contributed little to current published models [8, 35, 36].

It is currently difficult to identify those mothers with early-onset pre-eclampsia who are at increased risk of developing complications, and this risk cannot be graded. There is a need for a prognostic model to include the predictive role of more than one test results on the outcome with individualised risk assessment. Provision of individualised risk estimates for adverse maternal outcomes through the PREP study will help clinicians make suitable decisions after discussion with the parents.

Ideally, to develop a prediction model, we would like to observe outcomes in a cohort of women who receive no clinical management at all to be able to predict the likelihood of an adverse outcome independent of clinical management. Clearly, this is unethical and all women who present with pre-eclampsia receive clinical management, but such clinical decisions also affect maternal outcomes. Thus, in the development of our prediction model, we must recognise the importance of accounting for current clinical management; however, this is currently an under-researched methodological issue. By including effective treatment measures such as use of anti-hypertensives and magnesium sulphate predictor variables, and delivery before 34 weeks as a component of the composite outcome, we plan to negate some of the bias arising from treatment paradox in prediction models [23].

There is no obvious single outcome measurement that determines clinical management in early-onset pre-eclampsia. As the risk of more than one outcome needs evaluation simultaneously, we have chosen a composite measure consisting of several complications [37]. The composite outcomes are constructed by including those components whose underlying biology is similar [38].

To show that the PREP prognostic model is valuable, it is not sufficient to show that it successfully predicts outcome in the initial development data even after having it being internally validated. We need evidence that the model performs well for other (external) patients. The resulting geographical and domain validation will enable us to assess the prognostic performance and the generalisability of the model.

Our prediction model plans to use rigorous statistical methods to develop the model and assess accuracy; undertake a formal validation in external datasets (PIERS and PETRA); use unambiguous definitions of predictors and reproducible measurements using methods available in clinical practice; adjust for current clinical management; obtain input from patient focus groups and produce personalised estimates of risk, which enable patients and clinicians to make more informed decisions on management aspects like continuing the pregnancy or delivery of a preterm baby.

## Abbreviations

AUC: Area under the curve (*C*-statistic)
BP: Blood pressure
CEMACH: Confidential Enquiries into Maternal and Child Health
CMACE: Centre for Maternal and Child Enquiries
CTG: Cardiotocography
HELLP: Haemolysis, elevated liver enzymes, low platelets
PCR: Protein-creatinine ratio
PETRA: The Pre-eclampsia Eclampsia TRial Amsterdam
PIERS: Pre-eclampsia Integrated Estimate of RiSk for mothers
PREP: Prediction of Risks in Early onset Pre-eclampsia
RIND: Reversible ischemic neurological deficit
